# A comprehensive review of GABA in autism spectrum disorders: associations, mechanisms, and therapeutic implications

**DOI:** 10.3389/fpsyt.2025.1587432

**Published:** 2025-10-23

**Authors:** Peng Wang, Jin Sun

**Affiliations:** ^1^ Dalian Women and Children's Medical Group, Dalian, Liaoning, China; ^2^ Dalian Medical University, Dalian, Liaoning, China; ^3^ Department of Children's Health Care Medicine, Dalian Women and Children's Medical Group, Dalian, Liaoning, China

**Keywords:** autism, GABA, GABAergic neurons, GABAergic drugs, GABA levels

## Abstract

The etiology and pathogenesis of Autism Spectrum Disorder (ASD) are not yet clear. Gamma-aminobutyric acid (GABA), as an inhibitory neurotransmitter in the brain, is closely related to the pathogenesis of ASD. Animal models and clinical studies of ASD suggest that abnormalities in GABAergic neurons, signaling pathways, and related genes may play an important role in the pathogenesis of ASD, leading to abnormal levels of GABA in the blood and brain tissue of individuals with ASD. Additionally, GABAergic drugs have shown potential to improve ASD symptoms in animal models, but their efficacy and safety in clinical use still need further research. Therefore, this article reviews the relationship between GABA and ASD, as well as the related research on GABA levels and drug treatment, to further explore the pathogenesis of ASD and provide a theoretical basis for the diagnosis and treatment.

## 1 Introduction

Autism Spectrum Disorder (ASD) is a group of complex and severe neurodevelopmental disorders that typically begin in early childhood. It is characterized by social communication impairments, repetitive and stereotyped behaviors, restricted interests, and sensory perception abnormalities (1). Currently, the global prevalence of ASD is increasing rapidly, severely affecting the normal lives of children and adolescents. According to the latest research data released by the Centers for Disease Control and Prevention (CDC) in the United States in 2023, the prevalence of ASD among 8-year-old children in the United States is 27.6 per 1,000, equivalent to one ASD case in every 36 children, with the prevalence in boys being 3.8 times that in girls (2). The latest epidemiological study conducted in eight cities in China show that the prevalence of ASD nationwide to be 0.70% (3). It should be noted that there is a significant gender difference in the prevalence of ASD. Despite the high prevalence, the etiology and pathogenesis of ASD are still not fully understood. The etiology and pathogenesis of ASD mainly include genetic mutations and chromosomal structural variations, inflammatory responses and oxidative stress, immune dysfunction, and neurotransmitter imbalances (4, 5). The research on neurotransmitter abnormalities in children with ASD has primarily focused on glutamate, GABA, dopamine, serotonin, and oxytocin (6). Neurotransmitters are substances synthesized and released by presynaptic neurons, which can specifically act on receptors of postsynaptic neurons or effector cells to produce certain effects. The differences in the type, distribution, and concentration of neurotransmitters can have varying impacts on human cognition, emotions, and behaviors (7). In 2003, Rubenstein and Merzenich first proposed the Excitation/Inhibition (E/I) imbalance hypothesis. This hypothesis posits that higher brain functions rely on the precise balance of excitation and inhibition within the central nervous system, and disruption of this balance can lead to brain dysfunction (8). GABA signaling pathway serves as a key inhibitory regulatory system in the nervous system, encompassing the synthesis, release, transport, metabolism, and receptor binding of GABA. Any abnormality in these processes is closely associated with the pathogenesis of ASD. GABA may become the latest target for treatment. Animal research data shows that the expression of GABA levels is reduced in a rat model of ASD (9). Clinical studies have shown that children with ASD have significantly lower levels of GABA in the brain compared to typically developing children, which may be associated with dysfunction of GABAergic interneurons (10). In addition, studies have shown that the minicolumn structure in the cortex of people with ASDis abnormal, such as narrower minicolumns, increased number, and reduced spacing around neurons. These abnormalities may originate from asynchronous division of progenitor cells, leading to ectopic neuroblasts and dysmorphic cortical malformations (11). Furthermore, in people with ASD, impaired synaptic pruning results in insufficient long-range connections and excessive local connectivity. This could explain the difficulties people with ASD face in emotional signaling, attention shifting, and repetitive behaviors, ultimately contributing to E/I imbalance (12). This article mainly reviews the association, mechanism of action, drug therapy and clinical significance of GABA in ASD.

## 2 GABA plays a crucial role in the process of neurodevelopment

The dysfunction of the brain’s excitatory-inhibitory circuitry or the imbalance of excitatory and inhibitory neurotransmitters within this circuitry underlies many neuropsychiatric abnormalities. This imbalance is believed to be manifested in most brain regions, including the amygdala, cortex, and hippocampus, and plays a central role in several pathological conditions, such as Fragile X Syndrome (FXS), epilepsy, and ASD. The excitatory and inhibitory effects of neurons can be mediated by different neurotransmitters. When the levels of glutamate, an excitatory neurotransmitter, increase in the brain, or when the levels of GABA, an inhibitory neurotransmitter, decrease, the balance is disrupted. This disruption can lead to the manifestation of autistic symptoms (13). GABAergic system is the inhibitory neurotransmitter system in the central nervous system of vertebrates (14). GABA is the primary inhibitory amino acid neurotransmitter in the mammalian brain. It is synthesized from glutamate by the enzyme glutamic acid decarboxylase (GAD) through an α-decarboxylation reaction. GABA typically reduces neuronal excitability and regulates signal transmission between neurons, thereby modulating and inhibiting neural activity. GAD is the rate-limiting enzyme in GABA synthesis and exists in two isoforms in the brain: GAD65 and GAD67. GAD67 is encoded by the GAD1 gene and expressed in the neuronal soma or throughout the entire neuron, whereas GAD65 is encoded by the GAD2 gene and expressed at axon terminals. Consequently, GAD67 synthesizes GABA throughout the cell, controlling metabolic GABA production, while GAD65 synthesizes GABA at inhibitory synapses for vesicular release, participating in GABAergic synaptic transmission and plasticity (15, 16). In addition to its synthesis and release mechanisms, GABA is released from cells into the extracellular environment through exocytosis or reverse transport by GABA transporters (17). GABA primarily exerts its effects by acting on three specific types of receptors: GABAA, GABAB, and GABAC (14). GABA released into the synaptic cleft is primarily taken up back into presynaptic interneurons via the GABA transporter 1 (GAT1). A smaller portion is transported by GABA transporter 2 (GAT2), GABA transporter 3 (GAT3), and Betaine/GABA transporter 1 (BGT1). These transporters are responsible for shuttling GABA from the extracellular environment back into neurons (18, 19). GABA signaling pathway is a key regulatory system in the nervous system, which covers the synthesis, release, transport, metabolism and receptor binding of GABA. Decreased GABA synthesis (GAD1 abnormality), abnormal transport (GATS imbalance) and receptor dysfunction (GABA-A/B receptor variation) jointly weaken inhibitory signals, leading to over excitation of neural network. At the same time, the abnormal migration of GABAergic interneurons caused by the insufficient expression of GAD1 in the embryonic stage may permanently change the assembly of cortical circuits. GABA signaling pathway plays an important role in the pathogenesis and development of ASD (20).

## 3 Research on the association between GABA and ASD

### 3.1 The relationship between GABAergic interneurons abnormalities and ASD

**Figure 1:**
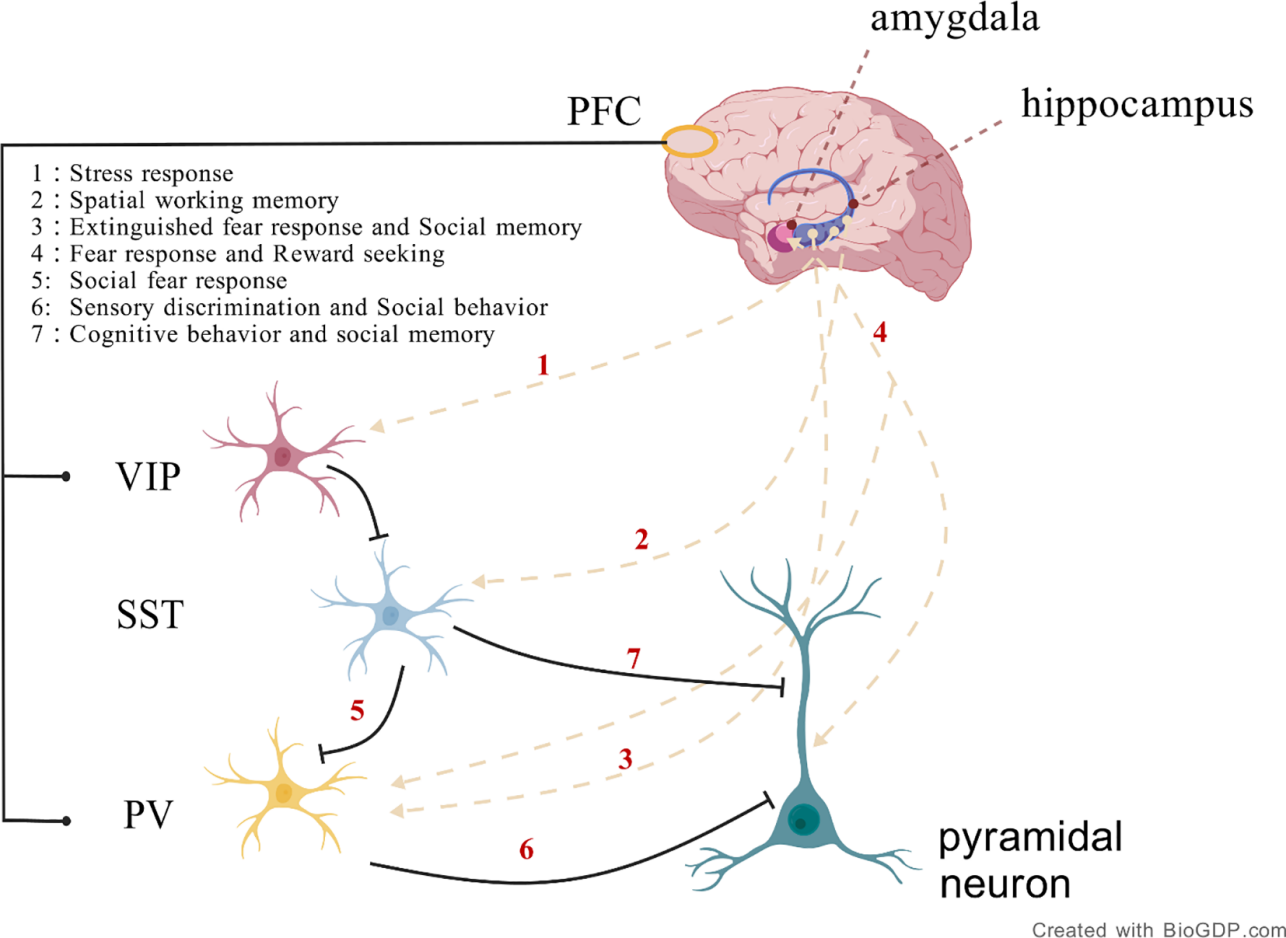
The connectivity between specific brain regions and inhibitory neuron types, including the connections between the hippocampus and prefrontal cortex involving PV, SST, and VIP neurons, plays a role in precisely regulating neural circuit activity during memory formation and consolidation. Additionally, the connection between the amygdala and PV neurons in the prefrontal cortex is involved in fear response and reward seeking. (Created with BioGDP.com).

In the mammalian brain, inhibitory GABAergic neurons are primarily interneurons, which include parvalbumin (PV)-expressing interneurons, somatostatin (SST)-expressing interneurons, vasoactive intestinal peptide (VIP)-expressing interneurons, and interneurons expressing the ionotropic serotonin receptor (5HT3a) (21). GABAergic interneurons primarily originate from the medial and caudal ganglionic eminences (MGE and CGE) in the ventral telencephalon during embryonic development. These interneurons migrate tangentially to distribute across multiple brain regions, including the prefrontal cortex, amygdala, and globus pallidus, which are closely related to the regulation of social behavior. Functional abnormalities of GABAergic neurons in these regions may constitute one of the pathological bases of ASD (**Figure 1**).

The MGE is the main source of cortical inhibitory interneurons, with its progenitor cells differentiating into PV and SST positive neurons. In contrast, the CGE gives rise to VIP and Reelin positive subtypes. These neurons migrate over long distances to enter cortical and subcortical regions, playing crucial roles in maintaining the brain’s excitation/inhibition balance and regulating synaptic plasticity. Their dysfunction is closely associated with neurodevelopmental disorders such as autism and schizophrenia (22).

The ventral pallidum (VP) receives GABAergic inputs from the nucleus accumbens and projects to the mediodorsal thalamic nucleus and prefrontal cortex, forming the “cortex-striatum-cortex” loop, which regulates motivated behavior and reward evaluation (23). The basolateral amygdala (BLA) modulates the projections from the mPFC through GABAergic interneurons, balancing self-interested and altruistic behaviors (24). Damage to the BLA leads to a significant reduction in altruistic behavior in social discounting tasks.Weakening of GABAergic inhibition in the amygdala-nucleus accumbens circuit results in social avoidance (25). Abnormal projections from PV positive GABAergic neurons in the CA1 region of the hippocampal-cortical circuit to the retrosplenial agranular cortex (RSA) can reversibly improve social deficits in ASD mouse models.

Evidence from single-cell transcriptomics indicates that in VIP interneurons of ASD patients’ brains, transcription factors such as Abelson Helper Integration Site 1 (AHI1) and synaptic genes like RAS-Associated Protein RAB3A (RAB3A) are downregulated. Moreover, SFARI genes are relatively enriched in VIP and SST interneurons, reflecting the disruption of gene expression regulatory networks in these interneurons in ASD. This disruption affects the inhibitory regulation between neurons, thereby disrupting the excitation/inhibition balance in the brain and promoting the emergence of core symptoms of ASD (26).

Futhermore, the aristaless-related homeobox (Arx) transcription factor, located on the X chromosome, has been implicated in a wide range of neurological disorders, including intellectual disability and epilepsy, as well as ASD. It is involved in the migration and differentiation of GABAergic interneurons in olfactory bulb (OB). In ARX knockout mice, the migration of GABAergic interneurons in OB was abnormal and the number decreased. Although the specific mechanism is not completely clear at present, from the perspective of its important regulatory role on GABAergic neurons, the abnormality of ARX may affect the normal development and function of GABAergic neurons, leading to the imbalance of brain neural circuits, thus causing ASD related symptoms (27).A substantial body of research has demonstrated that GABAergic interneurons play a crucial role in modulating synaptic plasticity, neuronal excitability, learning, and memory (28).

Dysfunction of GABAergic interneurons has been observed in animal models of ASD. In wildtype mice, optogenetic inhibition of GABAergic interneurons in the medial prefrontal cortex (mPFC) leads to cognitive deficits (29). Impairment of the three distinct subtypes of GABAergic interneurons has been implicated in the development of ASD. Specifically, the expression levels of somatostatin (SST)-expressing interneurons are reduced in the mPFC of ASD model mice, which may underlie their decreased social interest (30). Optogenetic enhancement of inhibitory parvalbumin (PV)-expressing interneurons rapidly rescues social deficits and hyperactivity in adult ASD model mice lacking CNTNAP2 (31). In FMR1 knockout mice, a decrease in the frequency of spontaneous excitatory postsynaptic currents (sEPSCs) is observed in PV interneurons, along with a reduction in synaptic number (32). A reduction in the number of PV+ neurons has been observed in human ASD brain slices (33). Transgenic mice with lower (PV^+/−^) or absent (PV^−/−^) expression exhibit the core ASD-like symptoms (34). PV deficiency leads to an increase in mitochondrial volume and density within somatic cells, and this change is positively correlated with the estimated PV concentration values in different Pvalb neuron subgroups in wild-type neurons. The increased dendritic length, branch number, and proximal dendritic thickness observed in PV-/- Pvalb neurons may be due to the increased mitochondrial density and length in the dendrites, suggesting that PV deficiency may be associated with the pathogenesis of ASD by altering neuronal morphology and function (35). Furthermore, researchers have found that the gene expression of vasoactive intestinal peptide (VIP)-expressing interneurons is downregulated in individuals with ASD compared to healthy controls, with the most significant differential expression, suggesting potential dysfunction of VIP interneurons in people with ASD (26).

In addition, Reelin is an extracellular matrix glycoprotein closely related to neural development, which is involved in the regulation of neuronal migration, synaptic plasticity and neural network formation (36).Reelin affects the migration and synaptic integration of GABAergic interneurons by regulating disabled-1 (Dab1) and PI3K/AKT pathways. In people with ASD, mutations or polymorphisms in the Reelin gene (RELN), such as at the rs736707 locus, can lead to reduced Reelin expression. This results in abnormal positioning of cortical GABAergic interneurons and a decrease in inhibitory synaptic density. Such deficits disrupt the temporal and spatial specificity of synaptic pruning, ultimately leading to the formation of overly connected neural networks. Additionally, GABAergic signaling inhibits the cAMP/PKA pathway within neurons via GABAB receptors. Under normal circumstances, this mechanism enhances Reelin expression, thereby maintaining the excitatory/inhibitory (E/I) balance in the brain. However, in people with ASD, abnormal GABA concentrations (e.g., fluctuations in plasma GABA levels) and defects in GABAB receptor function can impair this feedback mechanism. This not only exacerbates Reelin synthesis disorders but also disrupts neuronal migration, further interfering with neurodevelopment and network formation (37).

### 3.2 The relationship between GABAergic signaling pathway abnormalities and ASD

#### 3.2.1 The synthesis, release, transport, and metabolism of GABA

The biosynthesis of GABA primarily relies on the decarboxylation of glutamate catalyzed by GAD. In this process, the two isoforms of GAD, GAD65 and GAD67, play crucial roles. They are expressed in different locations within inhibitory neurons and fulfill distinct physiological functions. Studies have shown that the transcriptional level of GAD1 is decreased in people with ASD (38). In brain organoids cultured from induced pluripotent stem cells (iPSCs) of autism patients, the methylation level of CpG island in GAD1 promoter region is significantly increased (39). This methylation affects the region containing CTCF binding sites. CTCF is a transcription factor protein that regulates gene transcription by interacting with enhancers and promoters. Due to the increased methylation in the GAD1 promoter region, the binding of the transcriptional repressor MeCP2 is enhanced, resulting in downregulation of gene expression. Additionally, during early embryonic development, the methylation profile of the GAD1 gene becomes irregular, suggesting that epigenetic regulation may already be impaired during critical periods of neural development (40).Studies have shown that reduced expression of GAD65 or GAD67 in the central nervous system (CNS) can lead to ASD-like symptoms in mice. For example, in mice with a GAD67 haploinsufficiency, decreased expression of GAD67 and GABA levels in the brain are associated with increased anxiety and reduced social behavior, which are characteristic of ASD-like symptoms (41, 42). Post-mortem studies of ASD patient brains have revealed downregulation of both GAD65 and GAD67 in the superior temporal gyrus, potentially disrupting normal GABA synthesis in the brains of individuals with ASD (38). Therefore, decreased GABA levels in the CNS are implicated as a significant factor in the pathogenesis of ASD, and upregulation of GABA expression in the brain may improve ASD symptoms.

The release of GABA into the synaptic cleft is triggered by depolarization of the presynaptic membrane. GATS regulate the concentration of GABA in synaptic space through reuptake. The distribution and dysfunction of its subtype GAT1–3 affect the neurotransmitter homeostasis. GAT1 was mainly distributed in the presynaptic membrane of neurons and was responsible for 80% of GABA reuptake; GAT3 was located in glial cells and cleared the diffused GABA; GAT2 is highly expressed during development, which may be involved in the early formation of neural circuits. GABA is transported into presynaptic neurons via the GABA transporter type 1 (GAT-1), while the vesicular inhibitory amino acid transporter (VIAAT) utilizes the proton gradient generated by the vacuolar-type ATPase to transport GABA into synaptic vesicles. Subsequently, these GABA-containing vesicles release GABA into the extracellular space through a Ca2+-dependent membrane fusion mechanism. Extracellular GABA levels are not only regulated by the release mechanism but also by reuptake and metabolic processes.

The reuptake of GABA is mediated by GATs, with GAT-1 utilizing the electrochemical gradients of Na+ and Cl^-^ to transport extracellular GABA back into presynaptic and postsynaptic neurons, thereby facilitating the termination of GABAergic signaling. Once reuptaken into the cell, GABA is metabolized by GABA transaminase (GABA-T) and succinic semialdehyde dehydrogenase (SSADH) into succinate, which is then involved in the tricarboxylic acid (TCA) cycle. GAT-1 maintains the homeostasis of inhibitory neuronal microcircuits, and its functional or expression abnormalities are closely associated with the development of various neurological disorders, including autism, schizophrenia, and epilepsy (42, 43). The SLC6A1 gene encodes the GAT 1. Mutations in SLC6A1 result in partial or complete loss of GABA uptake function, causing ASD. This may be due to misfolding, retention in the endoplasmic reticulum, and degradation of mutant proteins (44).

#### 3.2.2 The Interaction between GABA and Its Receptors

GABA signaling transduction is mediated by membrane-bound receptors, primarily involving two major types: GABAA and GABAB receptors.

The GABAA receptor is a ligand-gated chloride (Cl^-^) channel, composed of five subunits arranged around a central pore, forming a heteropentameric transmembrane glycoprotein structure embedded in the neuronal membrane (45, 46). These subunits contain binding sites for various drugs, including GABA, benzodiazepines, and barbiturates (47). Binding of GABA to the GABAA receptor increases the permeability of the Cl^-^ channel, allowing Cl^-^ ions to flow into the cell along their concentration gradient. This influx of Cl^-^ leads to hyperpolarization of the neuron, thereby inhibiting neuronal excitability and reducing the propagation of nerve impulses. However, this signaling pathway does not always exert an inhibitory effect. Its polarity and efficacy are dynamically regulated by the electrochemical gradient of Cl^-^, which is modulated by the interplay between the Na^+^-K^+^-2Cl^-^ co-transporter 1 (NKCC1) and the K^+^-2Cl^-^ co-transporter 2 (KCC2). Under physiological conditions, immature neurons exhibit high expression of NKCC1 and low expression of KCC2, resulting in elevated intracellular Cl^-^ levels. Consequently, Cl^-^ efflux occurs upon GABAA receptor activation, leading to depolarization of the postsynaptic membrane and an excitatory effect of GABA. As neurons mature, KCC2 expression increases while NKCC1 expression decreases, lowering intracellular Cl^-^ levels. Under these conditions, Cl^-^ influx occurs upon GABAA receptor activation, resulting in hyperpolarization and the inhibitory effect of GABA. Thus, the functional balance between NKCC1 and KCC2 determines the balance of excitatory-inhibitory neural circuits. Studies have suggested that abnormalities in this polarity switch, caused by altered levels of NKCC1 and KCC2, may underlie the pathogenesis of various neurological disorders, including ASD (48–52). Treatment with selective NKCC1 inhibitors has been shown to improve social and repetitive behavioral deficits in ASD mouse models, restoring the NKCC1/KCC2 expression ratio to normal levels (53). GABRG3 gene deletion may reduce chloride ion transport, resulting in depolarization and an excitatory effect on neurotransmission (54). People with ASD exhibit reduced expression of GABAA receptor alpha5 subunits in the amygdala and nucleus accumbens, potentially affecting their emotion recognition and social interaction abilities (55).

GABAB receptors are G-protein coupled receptors (GPCRs) composed of the GABAB1 (GB1) and GABAB2 (GB2) subunits. On the other hand, they induce slow (hundreds of milliseconds) and sustained activity primarily through G i/o proteins by triggering signal transduction pathways and activating downstream effectors such as ion channels and adenylate cyclase. GABAB receptors function both presynaptically and postsynaptically; they either inhibit voltage-gated Ca 2+ channels to block neurotransmitter release or open G-protein gated inwardly rectifying K + (GIRK) channels to induce neuronal hyperpolarization. Since GABAB plays a central role in neurobiology, it is implicated in various neurological and psychiatric disorders, such as epilepsy, cognitive impairments, and ASD (56, 57). Studies have shown that downregulation of GABAB receptors leads to an imbalance in the excitatory-inhibitory neural circuits, resulting in ASD-like phenotypes in mice, such as reduced social interaction and increased repetitive and anxiety-related behaviors. Additionally, reduced densities of GABAB receptors have been found in the cingulate cortex and fusiform gyrus of people with ASD, which are closely related to face emotion recognition functions. These findings may indicate that these regions are one of the important neural bases for the social impairments in ASD, and also suggest that dysfunction of this key inhibitory receptor may lead to ASD-like functional deficits (58, 59). Animal studies report that the expression of the GABAB1 receptor in the prefrontal cortex (PFC) of valproic acid (VPA) induced ASD mouse models is significantly reduced. Using virus-mediated RNA interference to knock down the GABAB1 receptor in the PFC of mice resulted in ASD-related behaviors such as social interaction deficits, increased repetitive behaviors, anxiety, and cognitive impairments (58). The positive allosteric modulator GS39783 demonstrates anxiolytic effects in animal models, suggesting its potential as a target for ASD intervention (60).

#### 3.2.3 The relationship between GABA-related gene abnormalities and ASD

The genetic architecture of ASD is extremely complex. Approximately 600 to 1,200 genes and genomic regions have been identified as being associated with ASD. At least 5% of ASD cases are caused by single nucleotide polymorphisms (SNPs) in genes such as NLGN3, NLGN4, NRXN1, MECP2, SHANK3, FMR1, and UBE3A, with about 10% of these variants being copy number variations (CNVs) that disrupt protein-coding sequences (61–63).

##### 3.2.3.1 15q11-q13

The most common deletion/duplication syndrome associated with ASD phenotypes is the duplication syndrome of chromosome 15q11-q13.This region not only harbors the coding genes for GABA-A receptor β3 (GABRB3), α5 (GABRA5), and γ3 (GABRG3) subunits but also serves as a genetic vulnerability hotspot due to its enrichment in low-copy repeats (LCRs)Deletions in the BP1-BP3 breakpoint regions are associated with ASD syndromes (64, 65). Notably, these GABA-A receptor subunit genes are clustered on chromosome 15q12, forming a gene cluster comprising GABRB3, GABRA5, and GABRG3. Two single-nucleotide polymorphisms identified in the GABRG3 gene exhibit significant linkage disequilibrium with ASD, suggesting that GABRG3 or its neighboring genes may constitute genetic risk loci for ASD (66). Additionally, in specific genetic variation studies, higher frequencies of the “A” allele of GABRB3 (rs7180158), the “G” allele of GABRG3 (rs12901555), and the “T” allele of GABRA5 (rs35399885) were observed in people with ASD. The GA + AA genotypes (rs7180158) and CT + TT genotypes (rs35399885) were found to significantly increase the risk of ASD (64) These genetic variations may be associated with alterations in GABAergic signaling, which could contribute to the development of ASD symptoms such as social communication impairments and repetitive behaviors.

UBE3A gene is located on the q11-q13 region of human chromosome 15, encoding the E6AP protein, which is an E3 ubiquitin ligase protein involved in protein degradation and transcriptional regulation. Abnormalities in the UBE3A gene are closely associated with two neurodevelopmental disorders. When E6AP protein expression is absent, it leads to Angelman syndrome. Conversely, gene CNVs causing overexpression of E6AP are closely related to the onset of ASDs. Among these, maternal copy loss in the 15q11-q13 chromosomal region and maternal inheritance of UBE3A gene overexpression (such as in Dup15q syndrome) are both associated with the occurrence of autism (67, 68). The overexpression of UBE3A may disrupt the balance of protein degradation and transcriptional regulation, potentially contributing to the neuropathology of ASD. This genetic alteration could serve as a diagnostic biomarker and may provide a target for therapeutic strategies aimed at modulating UBE3A expression levels.

##### 3.2.3.2 FMR1

The *FMR1* gene (Fragile X Messenger Ribonucleoprotein 1) is a protein-coding gene. The protein it encodes, Fragile X Messenger Ribonucleoprotein (FMRP), is an RNA-binding protein that regulates synaptic function through the modulation of protein translation. It is implicated in the pathogenesis of Fragile X Syndrome (FXS) and ASD. Induced pluripotent stem cells derived from individuals with FXS have been shown to exhibit delayed GABA polarity conversion, reduced numbers of GABAergic inhibitory interneuron populations, and hyperexcitable membrane properties.

Animal studies have demonstrated that *Fmr1* knockout mice have fewer GABAergic synapses, reduced expression levels of GABAA receptors, and downregulation of GAD65 and GAD67 transcription. These mice also exhibit abnormal neurotransmission in brain regions associated with cognitive and social functions. At 4 weeks of age, GABAA receptor density in the cerebellum was significantly reduced, suggesting that GABAergic inhibitory transmission may be weakened during early developmental stages. This could potentially serve as a biomarker for early diagnosis of FXS. At 12 weeks of age, GABAA receptor density in the dorsal hippocampus was significantly increased, which may reflect compensatory enhancement of the GABAergic system in adulthood. In the prefrontal cortex and cerebellum, significant reductions in GABAA receptor density further indicate that GABAergic function is suppressed in these regions (69). In Fmr1 knockout mice, GABAergic inhibitory function in the CA1 region of the hippocampus was impaired, as evidenced by reduced amplitude and frequency of eIPSCs, spontaneous IPSCs, and miniature IPSCs, along with significantly decreased expression of α2, β1, and δ GABAA receptor subunits in the hippocampus, which is associated with behavioral and cognitive deficits. Levels of GABA, GABA-B receptors, GABAergic enzymes, and transport proteins in the brain during maturation of fragile X knockout mice showed no significant differences compared to wild-type mice (70). Studies using the GABA analog in Fmr1 knockout mice demonstrated corrections in hyperactivity and modulation of altered sensory-motor gating, measured as increased prepulse inhibition following acute treatment, by enhancing the activity of extrasynaptic GABA receptors, thereby improving anxiety, aggression, and repetitive behavioral abnormalities in FXS mice (71). Acute maternal pretreatment with the NKCC1 antagonist bumetanide was able to restore the excitation-inhibition switch in neonatal Fmr1 knockout mice and correct electrophysiological and behavioral phenotypes (72). This suggests the potential therapeutic value of targeting GABAergic systems for improving symptoms in FXS.

Additionally, reduced activity of PV expressing neurons in the V1 region of Fmr1 knockout mice may be associated with deficits in perceptual learning. Researchers used a chemogenetic strategy to activate PV neurons and found that this significantly improved learning speed and perceptual learning ability in Fmr1 knockout mice (73). *De novo* protein synthesis was elevated in hippocampal PV- and somatostatin-expressing inhibitory neurons. The cell-type-specific loss of Fmr1 in PV-expressing neurons led to anxiety-like behavior, impaired social behavior, and dysregulated *de novo* protein synthesis.

In contrast, the loss of Fmr1 in somatostatin-expressing neurons did not result in behavioral abnormalities or significantly affect *de novo* protein synthesis (74). A single dose of racemic baclofen significantly reduced gamma power in the EEG of Fmr1 knockout mice and improved inter-trial phase coherence (ITPC) deficits in response to temporally modulated auditory stimuli. Similarly, a single dose of RBAC in FXS patients resulted in a corresponding reduction in gamma power (75). These findings indicate that modulation of the GABA system may have therapeutic potential for improving specific neurophysiological and behavioral symptoms in FXS.

##### 3.2.3.3 MeCP2

Methyl-CpG binding protein 2 (MeCP2) is a chromatin-associated protein encoded by the *MECP2* gene. It plays a critical role in regulating gene transcription and expression in neurons and glial cells. MECP2 gene mutations lead to Rett syndrome, an X-linked neurodevelopmental disorder primarily affecting females. Approximately 60% of Rett syndrome patients meet the criteria for ASD, characterized by repetitive hand movements, social withdrawal, and loss of language communication.

Research has shown that restoring MECP2 expression solely in GABAergic neurons of male mice can significantly improve multiple disease features in a Rett syndrome mouse model, including ataxia, apraxia, and social abnormalities (76). Mecp2 mutant mice exhibit the elimination of GABA developmental shifts in CA3 pyramidal neurons at birth, with an increased glutamatergic/GABAergic postsynaptic current (PSC) ratio and hypersynchronous activity, as well as impaired metabotropic long-term depression (LTD). Administration of bumetanide, an NKCC1 chloride importer antagonist, can restore the GABA developmental shifts eliminated by Mecp2 mutations and reduce the increased glutamatergic/GABAergic PSC ratio (77). MECP2 expression levels are directly related to GABAA-A1R receptor expression, suggesting that MECP2 may influence neurotransmission by directly regulating the expression of GABAA-A1R receptors (78). In Mecp2-deficient mice, NTS neurons show significantly reduced amplitudes and frequencies of sIPSCs and mIPSCs, indicating impaired synaptic transmission, and decreased eIPSC amplitudes, though the paired-pulse ratio (PPR) remains unchanged, suggesting that postsynaptic mechanisms may contribute to the weakening of GABAergic inhibition. Additionally, in Mecp2 KO mice, GABA transporter GAT3 expression is increased in the hippocampus, along with corresponding increases in GAT3 currents in hippocampal astrocytes, and long-term administration of GAT3 antagonists can improve symptoms in Mecp2 KO mice (78). The disrupted beta synchrony observed in transgenic monkeys is likely a result of MECP2-induced GABAergic neuron dysfunction (79). Similar GABAergic signaling dysfunction and reduced beta synchrony at rest have been observed in people with ASD (80). These findings collectively underscore the importance of MECP2 in GABAergic signaling and its potential as a therapeutic target for both Rett syndrome and ASD.

##### 3.2.3.4 NLGNs

Neuroligins (NLGNs) are a family of transmembrane proteins encoded by the *NLGN* genes, located on the postsynaptic cell surface. They interact with neurexins (NRXNs) on the presynaptic cell surface to regulate synaptic differentiation, thereby contributing to the formation of either inhibitory or excitatory synapses (81).

Among the NLGN isoforms, NLGN1 is predominantly involved in excitatory synapse structure, transmission, and plasticity, but has minimal effects on inhibitory synapses.While its direct link to ASD remains unclear, alterations in NLGN1 expression may contribute to cognitive impairments through disruptions in synaptic plasticity and learning-related neural circuits (82).

In *Nlgn2* R215H mutant mice, inhibitory synaptic transmission is significantly reduced in the prefrontal cortex, accompanied by decreased levels of the GABAA receptor γ2 subunit and vesicular transporters. This leads to an E/I imbalance and abnormal gamma oscillations in the prefrontal cortex of these mice (83). This gamma oscillation is a high-frequency (approximately 30–100 Hz) electrocortical wave oscillation, typically associated with cognitive functions and sensory information processing. In people with ASD, the abnormality of this gamma oscillation is often regarded as an expression of E/I imbalance, reflecting a defect in the function of cortical GABAergic interneurons. Overexpression of NLGN2 in adult mice leads to increased localization of GABAergic synaptic proteins at presynaptic and postsynaptic sites, associated with deficits in hippocampus-dependent behaviors and memory formation (84). These findings suggest that gamma oscillations may serve as a potential biomarker for assessing E/I balance and treatment response in ASD.

The Nlgn3 gene contains two splice insertion sites (A1 and A2) that can yield four NLGN3 splice isoforms (Nlgn3Δ, A1, A2, and A1A2). NLGN3 protein in the hippocampus differentially regulates inhibitory synaptic transmission in a splicing isoform-dependent manner. Overexpression of NLGN3Δ or 3A2 increases evoked inhibitory postsynaptic currents (IPSCs), while overexpression of NLGN3A1 or 3A1A2 leads to a reduction in IPSC amplitude. Although all isoforms promote excitatory synaptic transmission, their subcellular localization varies significantly, with NLGN3Δ diffusely distributed in dendrites and NLGN3A1A2 enriched in spines, revealing that splicing-dependent isoform distribution specificity is a key molecular mechanism for bidirectional regulation of inhibitory synapses (85). Mice with the NLGN3-R451C mutation exhibit social deficits and behavioral problems, with increased excitatory synaptic transmission in the CA1 region of the hippocampus and enhanced inhibitory synaptic transmission in the 2/3 layers of the somatosensory cortex (86).Enhanced inhibitory synaptic transmission in Purkinje cells is manifested as an increase in the amplitude of mIPSCs, with no significant change in frequency (87). These regional differences in synaptic dysfunction may underlie the heterogeneous behavioral phenotypes in ASD and support the development of region-targeted therapies.

Research found that a decreased network response to stimulation in both excitatory and inhibitory circuits of the sensory cortex of Nlgn4 knock-out animals as compared to wild-type controls, and a decreased excitation-inhibition ratio, suggesting that Nlgn4 is involved in the regulation of excitatory and inhibitory circuits and contributes to a balanced circuit response to stimulation (88).Another study reports, Nlgn4 knockout disrupts GABAergic synaptic transmission and oscillatory activity in the hippocampus and causes deficits in social interaction. In L4 spiny stellate neurons, Nlgn4-KO results in a significant decrease in the frequency of glutamatergic and GABAergic miniature currents (mEPSCs and mIPSCs). The absence of Nlgn4 weakens glutamatergic and GABAergic synaptic transmission, tilting the excitation/inhibition balance toward inhibition (89). Nlgn4 gene deletion specifically alters the structure and function of perisomatic inhibitory synapses on PV-positive interneurons in the hippocampal CA3 region, significantly disrupting the generation of gamma oscillations. This may lead to overall network activity interruption through cumulative effects, especially abnormal synchronous oscillations such as gamma rhythms. This mechanism may represent a key pathway for cognitive impairment in individuals with autism (90). Given the strong association between gamma oscillations and cognitive processing, such disruptions may contribute to the cognitive impairments seen in ASD, making NLGN4 a promising target for interventions aimed at restoring synchronized network activity.

##### 3.2.3.5 SHANK

The SHANK family belongs to synaptic scaffold proteins, including SHANK1, SHANK2, and SHANK3 genes, which are located in the postsynaptic density of excitatory glutamatergic synapses. These proteins can interact with neuroligins (NLGNs) and neurexins (NRXNs) to promote the formation of a complex involving N-methyl-D-aspartate receptors (NMDARs) and NLGN-NRXN-SHANK (91). This complex is essential for synapse formation, stabilization, and functional regulation, and its disruption has been strongly implicated in neurodevelopmental disorders such as ASD.

Among the SHANK isoforms, the SHANK3 gene is most closely associated with ASD and related neurodevelopmental conditions such as Phelan-McDermid syndrome. Shank3B knockout (Shank3B −/−) mice exhibit hypersensitivity to tactile stimuli, which correlates closely with increased spontaneous discharge and stimulus-evoked activity in pyramidal neurons, as well as reduced activity in interneurons, within the primary somatosensory cortex (vS1). Further studies using virus-mediated gene knockout and chemogenetic approaches have confirmed that knocking out the Shank3 gene specifically in interneurons is sufficient to cause hyperexcitability in pyramidal neurons and sensory hypersensitivity (92). Additionally, in Shank3αβ−/− mice, the expression levels of glutamate receptors are reduced in the striatum and thalamus, although no changes in the cell surface expression of GABAA receptor α1 subunits were observed in the cortex, striatum, thalamus, or cerebellum (93). Studies have found a trend of reduced glutamate and glutamine levels in the striatum of Shank3 KO mice. The correlation between striatal glutamate levels and the severity of social deficits aligns with the role of the striatum in social behavior. Abnormalities in the glutamate/GABA system within the corticostriatal circuit may represent a key pathological mechanism in ASD and could be associated with alterations in the neuroligin-neurexin signaling complex (94, 95). These findings suggest that glutamate/GABA imbalance, particularly within the corticostriatal pathway, may serve as a key pathophysiological mechanism and potential biomarker in SHANK3-associated ASD. Additionally, the absence of Shank3 in PV-positive neurons leads to repetitive behaviors, memory impairments, and motor coordination issues in mice. Using local field potential (LFP) and visual evoked potential (VEP) recordings, it was discovered that Shank3-deficient mice exhibit impaired visual cortical gain control, manifesting as a limited dynamic response range to visual stimuli. This alteration can be rescued by enhancing GABAA receptor activity. Treatment with ganaxolone, a positive allosteric modulator of GABAA receptors, improves behavioral deficits in mice with specific Shank3 gene knockout in PV-positive neurons (96).

Shank1 mutant mice exhibit impaired inhibitory synaptic output in hippocampal CA1 pyramidal neurons, leading to an imbalance between excitatory and inhibitory transmission (97). *Shank2* knockout mice show decreased expression levels of Gabra2 and GABAR-mediated inhibitory signaling (98). Shank1 -/- and Shank3B -/- mice show reduced PV expression in the mPFC, somatosensory cortex, and striatum. Shank2 knockout mice (e6–7 KO) exhibit decreased expression levels of Gabra2 and GABA receptor-mediated inhibitory signaling, which can be reversed by treatment with allosteric modulators of GABA-ARs (99, 100).

##### 3.2.3.6 CNTNAP2 and En2

Contactin-associated protein 2 (CNTNAP2) encodes a cell adhesion molecule belonging to the neurexin family. Mice with a knockout of CNTNAP2 exhibit autism-related behaviors, including social and communication impairments, repetitive behaviors,and seizures. These findings align with clinical observations in humans, where biallelic CNTNAP2 variants are linked to severe cognitive impairment, epilepsy, and behavioral abnormalities, underscoring its role in ASD pathogenesis (101).

The loss of CNTNAP2 leads to synaptic changes *in vitro*, reduced inhibitory and axonal excitability defects in acute hippocampal slices (102, 103). CNTNAP2 mutations may be associated with abnormal behaviors through alterations in synaptic neurotransmission, functional connectivity, and neuronal network activity. Recent studies have demonstrated that Cntnap2 KO mice exhibit reduced synaptic inputs to mPFC L2/3 pyramidal neurons, accompanied by a decrease in spines and synapses. These changes disrupt network activity, with mPFC cells showing reduced phase locking and altered preferred phases, thereby slowing *in vivo* mPFC local field potential (LFP) oscillations, which may underlie autism-related phenotypes (104). Another study investigated the developmental changes in tonic and phasic inhibition using the visual cortex as a model in Cntnap2 KO mice, revealing that Cntnap2 deficiency causes developmental disruption of GABA neurotransmission, particularly a reduction in tonic inhibition. This suggests that GABAARs containing δ subunits may be useful targets for therapeutic interventions in autism (105). Using human brain organoid models derived from induced pluripotent stem cells (iPSCs) with targeted knockout of the CNTNAP2 gene, which is associated with autism spectrum disorder (ASD), it was revealed that CNTNAP2-deficient brain organoids exhibit abnormalities such as accelerated cell cycle, increased cortical folding, disrupted glutamatergic/GABAergic synaptic pathways, and upregulation of AKT/mTOR signaling (106). The AKT/mTOR hyperactivation in these organoids suggests that mTOR inhibitors (e.g., rapamycin analogs) may ameliorate synaptic imbalances, warranting clinical exploration in ASD subtypes with CNTNAP2 mutations.

There is evidence suggesting that the homeodomain transcription factor Engrailed2 (En2) appears to be a susceptibility locus for ASD (107).

EN2 is primarily found in postnatal GABAergic neurons in the mouse hippocampus and cortex. Its role in GABAergic neuron development implicates it in ASD’s neurodevelopmental origins, particularly in sensory and cognitive processing deficits linked to GABAergic circuit imbalances. Research indicates that the sustained high expression of EN2 during prenatal and early postnatal periods contributes to autism by affecting the maturation of cerebellar purkinje cells (108). This developmental window may represent a critical period for interventions targeting cerebellar circuitry disruptions linked to ASD motor and cognitive symptoms.In En2 knockout mouse models, both young (P30) and adult En2-/- mice exhibit reduced expression of GABAergic interneuron markers parvalbumin (PV) and somatostatin (SST) in the forebrain. Additionally, the Gabrb3 is upregulated at P30 but downregulated in the adult hippocampus. The dynamic expression of Gabrb3 suggests it could serve as a stage-specific biomarker for early postnatal GABAergic dysfunction in ASD. This confirms that GABAergic neurotransmission deficits are present as early as the early postnatal period in the En2 mouse model of ASD, highlighting the potential role of neurodevelopmental defects in GABAergic interneurons in the pathogenesis of ASD (109). Evidence from *in vitro* studies reveals that En2 increases the complexity of GABAergic neuron dendritic trees, reduces the number of mature synapses, and decreases the area of postsynaptic density (110). These findings suggest that restoring En2-regulated synaptic pruning or enhancing GABAergic synapse formation could rescue circuit imbalances in En2-associated ASD.

##### 3.2.3.7 DLX and MET

In addition, genes related to GABA function play a significant role in the pathogenesis of ASD. The cell migration gene family DLX (such as DLX1, DLX2, DLX5/6) are homeodomain transcription factors that primarily govern the generation and migration of GABAergic interneurons in the forebrain. They not only directly activate the expression of GAD65/67 and VGAT, ensuring the GABAergic properties of interneurons, but also regulate genes such as Arx, guiding interneurons to migrate from the ventral forebrain to the cortex and form inhibitory circuits. Mice with DLX1 knockout exhibit age-dependent reductions in interneurons, weakened GABAergic synaptic currents, and eventually develop epilepsy and social abnormalities (111). DLX genes are involved in the formation of auditory organs, which may explain sensory processing abnormalities in people with ASD (112). DLX collaborates with mammalian achaete-scute homologue-1 (MASH1) to regulate the fate of progenitor cells, with approximately 65% of human neocortical GABAergic neurons dependent on DLX1/2 expression. Dysregulation of DLX function leads to insufficient cortical inhibitory input, disrupting the stability of local circuits (113). Restoring DLX-mediated GABAergic neuron differentiation or enhancing GABA signaling (e.g., via GABAA receptor agonists) could potentially rescue circuit imbalances in DLX-related ASD subtypes. Alleles such as s4519482 (DLX1) and rs743605 (DLX2) show significant over-transmission in ASD families, with a stronger combined risk effect (114). These variants may serve as genetic biomarkers for early screening and stratification of ASD subtypes with GABAergic dysfunction.

The tyrosine kinase receptor gene MET encodes the hepatocyte growth factor receptor, participating in neurodevelopment, regulating dendritic branching, synaptic plasticity, and cortico-striatal connections, and also influencing immune regulation and gastrointestinal repair, being associated with comorbidities in ASD (115). MET’s dual role in brain development and immune-gut crosstalk suggests it underpins both core ASD symptoms and systemic comorbidities (e.g., gastrointestinal dysfunction), making it a key node for multi-targeted therapies. The rs1858830-C allele is elevated by 2.27 times in ASD, reducing MET transcriptional activity by 50% and disrupting the binding of transcription factor complexes (116). Post-mortem cortical tissue from people with ASDshows significantly reduced MET protein levels, accompanied by dysregulation of HGF-MET co-expression, suggesting disrupted signaling pathways (117). These findings implicate MET as a therapeutic target, with strategies like MET agonists or microbiota-based interventions (to modulate gut-brain axis signaling) offering potential benefits. Therefore, the overall results suggest that GABA-related gene polymorphisms are sufficient to induce autism behavioral phenotypes. Whether rescuing GABA-related gene defects through genetic methods can improve behavior is worthy of further exploration (102, 109).

**Table 1** summarizes GABA signaling-related genes closely associated with ASD and the research findings related to them. These genes play a crucial role in the development, signal transduction, and synaptogenesis of GABAergic neurons, and their aberrant expression or dysfunction is closely linked to the pathogenesis of ASD.

**Table 1:** Related genes, functions, and their relevance to ASD.

Gene	Function	Mechanism	Reference
GAD1	Glutamate decarboxylase67 (GAD67), the rate-limiting enzyme for GABA synthesis	Downregulation of expression leads to reduced GABA synthesis, resulting in an imbalance of excitation/inhibition (E/I)	(38)
GAT1	GABA reuptake transporter	Impaired GABA clearance in the synaptic cleft leads to delayed termination of the inhibitory signal	(44)
GABRA5	GABAA receptor α5 subunit	Decreased density of α5 subtype receptors affects the inhibitory control of the prefrontal cortex and the ability to recognize facial emotions	(55)
GABRB3	GABAA receptor β3 subunit	Copy number changes in receptor subunits caused by duplication/deletion at 15q11–13 region, affecting receptor density and function	(64)
GABRG3	GABAA receptor γ3 subunit	Low-copy repeat sequences and allelic mutations at the GABRG3 locus are associated with ASD	(64, 65)
GABBR1	GABAB receptor subunit 1	Downregulation of GABBR1 can induce central ASD-related phenotypes in mice	(58)
UBE3A	Maternally expressed gene involved in transcriptional regulation	Duplication of the UBE3A and GABAA genes in the 15q11.2-13.1 region induces ASD-like phenotypes	(68)
FMR1	Encodes FMRP, a protein that regulates protein translation and synaptic function	Individuals with FXSand Fmr1 knockout mice exhibit abnormalities in GABAergic function, including a reduced number of GABAergic interneurons, decreased expression of GABAA receptors, and impaired inhibitory synaptic function. These abnormalities are associated with behavioral and cognitive deficits	(69–75)
MeCP2	Encodes a chromatin structural protein that regulates gene transcription	MeCP2 deficiency leads to reduced GABAergic synapse numbers and impaired inhibitory function	(76–80)
NLGNs	Postsynaptic transmembrane proteins that influence synapse formation	NLGN2 is involved in cognitive function, NLGN3 affects hippocampal dependent behavior and memory formation, and NLGN4 is involved in social behavior	(84, 85, 87, 89, 90, 92)
SHANK	Scaffolding proteins involved in synapse formation	SHANK1 regulates inhibitory synaptic function, SHANK2 influences GABA receptor-mediated inhibitory signaling, SHANK3 is closely associated with ASD, regulating glutamatergic and GABAergic synaptic functions, and affecting sensory hypersensitivity, social behavior, and neural circuit balance	(83, 93, 94, 96–98)
CNTNAP2, EN2	CNTNAP2 encodes a cell adhesion molecule, and EN2 encodes a homeodomain transcription factor	Knockout mice for these genes exhibit ASD-like behaviors	(102–109)
DLX	Homeobox transcription factors that regulate the generation and migration of GABAergic interneurons	Dysfunction or knockout of DLX genes can lead to insufficient cortical inhibitory input, disrupt neural network balance, and increase the risk of ASD	(113, 114)
MET	Tyrosine kinase receptor involved in neurodevelopment	MET is involved in neural development and synaptic plasticity, regulating immunity and gastrointestinal function. Functional abnormalities affect neural excitability and are closely related to the pathological mechanism of ASD	(115 **;** 116 **;** 117)

## 4 GABA levels and pharmacotherapy in ASD clinical applications

### 4.1 Abnormal GABA levels in the blood of ASD patient

A study from Egypt shows that the serum GABA levels in children with ASD are significantly reduced, as are the levels of GABAB receptors, while the serum glutamate levels are significantly increased. Additionally, the serum potassium, calcium, and zinc levels in children with ASD are also significantly lower than those in the control group. GABAB and zinc are extremely negatively correlated with stereotyped and repetitive behaviors (118). However, contrary to the findings in Egypt, a study by Indian scholars reported higher plasma GABA levels in children with ASD compared to controls (119). Furthermore, Al-Otaish et al. investigated serum GABA, glutamate (Glu), and the GABA/Glu ratio in children with ASD and found that plasma GABA, Glu, and the GABA/Glu ratio were significantly elevated in children with ASD (120). A case-control study by Afaf El-Ansary et al. demonstrated that, compared to controls, individuals with ASD exhibit higher levels of GABA and lower glutamate/GABA ratios. Individuals with ASD showed significantly elevated glutamate concentrations, indicative of glutamatergic excitotoxicity. Despite increased GABA levels, the greater rise in glutamate results in a reduced glutamate/GABA ratio, suggesting relative impairment of inhibitory function, such as decreased density of GABAA receptors, reduced expression of GABAA and GABAB receptor subunits in key brain regions, and downregulated expression of the GABA-synthesizing enzymes GAD65 and GAD67.Furthermore, elevated plasma GABA levels are positively correlated with glutamate and the glutamate/GABA ratio. The marked increase in plasma GABA appears to stem from increased availability of its precursor glutamate, indicating that peripheral GABA elevation reflects substrate excess rather than enhanced central inhibitory tone (121). Collectively, the inconsistent findings regarding plasma GABA levels in ASD may arise from the fact that GABA function is regulated by multiple mechanisms. The physiological effects of GABA depend not only on its concentration but also on factors such as receptor density and subunit composition, chloride ion gradients, and presynaptic release efficacy. Additionally, methodological limitations should not be overlooked. These include insufficient sample sizes, which may compromise statistical power and lead to inaccurate effect size estimation; variability in GABA measurement techniques—such as differences in ELISA kits—that can affect result comparability; inadequate control for demographic confounders, potentially introducing confounding bias; and limitations in analytical approaches that may hinder comprehensive interpretation of complex neurochemical relationships.Therefore, the effectiveness and reliability of GABA as a potential clinical diagnostic biomarker in the blood of children with ASD still require further validation and exploration through additional research.

### 4.2 Abnormal GABA levels in the brain tissue of ASD patients

Postmortem examination, single-photon emission computed tomography (SPECT), and magnetic resonance spectroscopy (MRS) have been employed to conduct in-depth studies on the brains of ASD patients. These studies have revealed that GABA levels in the cerebellum decreased by 50% overall, with a reduction in the number of GABAergic Purkinje cells in the cerebellar cortex. Significant decreases were observed in GABBR1, GABBR2, GABRA1, and GABRB3. In the parietal cortex, GABRA1, GABRA2, GABAR3, and GABRB3 were significantly reduced, and in the frontal cortex, GABRA1 was significantly decreased (122–124). In the hippocampal region, the density of GABAA receptors is also decreased. The cingulate cortex, a region crucial for social interaction and communication, exhibits a significant decrease in the density of GABAA receptors and their benzodiazepine binding sites (125), alongside a notable reduction in GABAB receptors, which is negatively correlated with social impairment scores (126). SPECT imaging has shown reduced expression of GABAA receptors in the bilateral frontal cortices of children with ASD. Positron emission tomography (PET) has identified lower GABAA receptor levels in the limbic system, including the nucleus accumbens, amygdala, and hippocampus, in high-functioning adult ASD patients compared to healthy controls (55, 127).

Additionally, using PET, it was found that the binding potential of GABA receptors in the brains of FXS patients showed a significant average decrease of 10%, with a 17% reduction in the thalamus region. This is the first study to directly demonstrate reduced GABA receptor availability in human FXS patients, supporting the hypothesis that GABA receptors could be a potential target for FXS drug therapy (128).

Proton magnetic resonance spectroscopy (1H-MRS) has detected reduced GABA concentrations in the frontal and motor cortices, cerebellum, anterior cingulate cortex, and sensorimotor regions of ASD patients, along with decreased ratios of GABA/glutamate, N-acetylaspartate/creatine, GABA/creatine, and glutamate/creatine. Elevated glutamate levels have also been reported (55, 127, 129–131). However, some studies have found increased GABA concentrations and GABA/creatine ratios in the left dorsolateral prefrontal cortex of adult ASD patients, which may support the excitation/inhibition (E/I) imbalance hypothesis, suggesting enhanced inhibitory function in this region (132, 133). It is conceivable that in ASD, neuronal hyperexcitation—consistent with the excitatory-inhibitory imbalance hypothesis—triggers compensatory excessive inhibition in specific brain regions, which may be reflected by elevated GABA concentrations. This is consistent with the theory of local area network inhibition, which posits that excessive or unstable excitatory activity can induce homeostatic over-inhibition, thereby contributing to clinical symptoms—whose precise manifestations depend on the neural networks involved. Thus, elevated GABA levels may represent a compensatory mechanism in response to altered GABAergic metabolism elsewhere, such as abnormalities in GABA receptor function (133, 134). Additionally, lower levels of N-acetylaspartate (NAA) and GABA/creatine ratios have been observed in the temporal cortex of ASD patients.

A 3T single-voxel HERMES MRS analysis showed no significant differences in glutathione (GSH) and GABA levels between ASD and typically developing groups in the primary motor cortex, thalamus, and medial prefrontal cortex (135). Autoradiography revealed that autistic patients exhibited a significant decrease in the number of GABAA receptors and benzodiazepine binding sites in the fusiform gyrus (FFG) and posterior cingulate cortex (PCC), accompanied by an increased binding affinity at the surface of GABAA receptors. These alterations in the FFG and PCC may influence facial and emotional processing, potentially contributing to the social deficits observed in autistic individuals (136). The average density of GABAA receptors in the granular upper and lower layers of the anterior cingulate cortex (ACC), as well as the density of benzodiazepine binding sites in the granular upper and lower layers, was significantly reduced (125).

Notably, GABA concentrations tend to increase during early development, begin to decline between 3 and 6 months postnatally, and stabilize in adulthood. These findings suggest that GABA and its metabolite levels and ratios may serve as potential targets for the clinical diagnosis and treatment of ASD (137).

### 4.3 Comorbidities related to ASD

Disruption of the GABAergic system is a core pathological mechanism underlying various ASD syndromes, such as FXS, Rett syndrome, and Dup15q, manifesting as E/I imbalance, abnormal gene expression, and defects in interneuron development. The comorbidity spectrum encompasses epilepsy, anxiety, sensory abnormalities, and other multisystem disorders, necessitating comprehensive strategies targeting GABA receptors, ion channel regulation, and the gut-brain axis (100). In FXS, CGG repeat expansions in the FMR1 gene lead to the loss of FMRP, impairing the translation of GABAA receptor mRNAs, particularly the GABAA α5 subunit, reducing receptor density in the amygdala and nucleus accumbens, resulting in delayed language development and autistic-like behaviors (138). Rett syndrome, caused by MECP2 gene mutations, disrupts GABAergic interneuron development, lowering GABA concentrations in the hippocampus and prefrontal cortex, leading to developmental regression, such as loss of language and motor skills, and stereotypic hand movements like hand-wringing (77). Epilepsy arises from insufficient GABAergic inhibition, causing hyper-synchronized neuronal firing. FXS, Rett syndrome, and Dup15q patients exhibit epilepsy at rates as high as 80% (139). Anxiety and ADHD stem from inadequate GABAergic inhibition in the prefrontal and limbic systems, disrupting emotional regulation. FXS patients have ADHD, and experience severe anxiety (140).

### 4.4 The application of GABAergic drugs in the treatment of ASD

#### 4.4.1 GABA receptor agonists

In the clinical treatment of ASD, the application of GABA receptor agonists is becoming increasingly widespread. Recent animal experiments have shown that intracortical injection of the GABAA receptor agonist clonazepam and the GABAB receptor agonist baclofen into the medial prefrontal cortex of ASD model mice induced by VPA can effectively alleviate ASD-like behaviors. The use of either GABAA or GABAB receptor agonists alone can partially reverse VPA-induced ASD behaviors, while the combination of clonazepam and baclofen can more effectively improve social deficits, enhance recognition memory, and reduce repetitive behaviors (141). Furthermore, in the maternal immune activation (MIA) mouse model induced by poly(I:C), low-dose clonazepam treatment increased inhibitory neurotransmission and reduced neural activity in the prefrontal cortex of offspring, which helped prevent social deficits, anxiety, and depression-like behaviors (142). Studies have also shown that long-term oral administration of baclofen can improve ASD-like behaviors in VPA-exposed mice after weaning, and prenatal intervention in offspring can also improve ASD-like behaviors, demonstrating the potential of early intervention with GABAB receptor agonists in treating ASD (143). Fmr1KO mice exhibit increased power of high-frequency gamma oscillations under auditory stimulation, along with impaired working memory, reduced sociability, and anxiety-like behavior.

Baclofen treatment normalizes the abnormal auditory-induced gamma oscillations, working memory, and anxiety-related behaviors but does not improve the deficit in sociability (144). A recent 10-week randomized, double-blind, placebo-controlled trial by Mahdavinasab et al. investigated baclofen as an adjunct to risperidone in children with ASD and moderate-to-severe irritability. The study reported that the combination of baclofen and risperidone led to significantly greater improvement in hyperactivity symptoms compared to risperidone plus placebo, with no significant differences observed for other domains such as irritability or social withdrawal. Despite employing a rigorous study design and demonstrating potential to significantly ameliorate hyperactive symptoms in children with ASD, the findings are constrained by several limitations. These include a limited sample size (N = 64), the lack of statistically significant differences in primary outcome measures, a narrow efficacy profile, a brief treatment duration, and the inability to isolate independent therapeutic effects due to its adjuvant nature. Additionally, the long-term safety profiles remain undetermined. These limitations collectively render the evidence insufficient to support routine clinical application. Therefore, further investigation is warranted through large-scale, extended-duration, monotherapy-focused Phase III trials to corroborate these findings and establish definitive efficacy and safety parameters (145).

Arbaclofen, the R-enantiomer of baclofen, has been shown to significantly reduce repetitive grooming behaviors in Fmr1 knockout mice following acute injection, and systemic treatment improved sensory filtering abilities in Cntnap2 knockout rats, suppressing excessive acoustic startle responses (146). Meanwhile, Arbaclofen can correct the social deficits in mice while also normalizing excessive baseline protein synthesis, abnormal AMPA receptor internalization, and increased dendritic spine density (147, 148). Additionally, arbaclofen administration improved ASD-like symptoms in mice prenatally exposed to VPA, suggesting that children with a history of prenatal VPA exposure may also benefit from GABAB receptor agonist treatment (143). While early open-label and phase 2 trials suggested potential benefits of arbaclofen in children with ASD, subsequent phase 3 randomized controlled trials failed to demonstrate significant efficacy over placebo on primary outcomes, despite favorable safety and tolerability profiles. These inconsistent findings highlight the challenges in translating preliminary signals into robust clinical effects and indicate that, at present, there is insufficient evidence to support the routine use of arbaclofen in ASD (149–151).In another randomized, double-blind, placebo-controlled trial in children with FXS, ganaxolone—a GABAA receptor positive allosteric modulator with a favorable safety profile—failed to show significant improvement over placebo on the primary outcome (CGI-I) or the key secondary outcome (PARS-R) in the overall population. However, *post hoc* analyses suggested potential benefits in prespecified subgroups, including those with higher baseline anxiety or lower cognitive functioning, warranting further investigation in these subpopulations. Given that FXS is a monogenic cause of ASD, these findings highlight both the challenges of translating GABAergic modulation into broad clinical efficacy and the importance of patient stratification in future trials of targeted therapies for neurodevelopmental disorders (152). Gaboxadol, a δ-subunit-selective, extrasynaptic GABAA receptor agonist that rescues FXS-related behaviors in Fmr1 KO mice, such as aggression, anxiety, hyperactivity, and stereotypies (153). A 12-week phase 2a trial reported that 60% of gaboxadol-treated individuals with fragile X syndrome (FXS) were CGI-I responders. However, due to the absence of a placebo control and small sample size, this result reflects only a preliminary signal and does not constitute robust evidence for efficacy. As such, it does not support clinical recommendations for gaboxadol in FXS or broader ASD populations (154).

Additionally, GABAA receptor subunit-selective modulators (the α_2,3_-subunit-selective positive allosteric modulator L-838,417) are particularly effective in improving autism-like behaviors in BTBR mice (155). Currently, no GABA receptor agonists have been specifically designed for the treatment of ASD, and their efficacy and safety in ASD treatment still require further validation through additional studies. GABA receptor agonists hold promise as a potential new therapeutic option for ASD.

#### 4.4.2 Homotaurine

Homotaurine (3-aminopropanesulfonic acid) is a natural amino acid found in algae. Its molecular structure is derived from taurine with the addition of a methylene group, making it a structural analog of GABA. The carboxyl group of homotaurine is replaced by a sulfonic acid group, endowing it with chemical properties extremely similar to those of GABA. This allows homotaurine to act as an agonist of the GABAA receptor, exerting inhibitory effects on the central nervous system (CNS). Homotaurine can cross the blood-brain barrier, exhibits high affinity for GABAA receptors, and has a longer plasma half-life compared to GABA. These characteristics make it a potential candidate for the treatment of various CNS disorders, including Alzheimer’s disease, multiple sclerosis, and epilepsy (156, 157). Rubal et al. demonstrated that treatment with homotaurine in rats with VPA-induced ASD improved social and anxiety-related behaviors. Additionally, homotaurine reduced the increase in oxidative stress biomarkers and inflammatory cytokines, such as IL-1β, IL-6, and TNF-α levels, caused by VPA in rats, decreased pyknosis in the prefrontal cortex, and restored the size and number of Purkinje cells in the cerebellum and regulated the dysregulated expression of GAD67 due to VPA exposure (158). In summary, experimental studies not only highlight the therapeutic potential of homotaurine for ASD but also support its candidacy as a treatment option. Further preclinical and clinical research is necessary to validate its efficacy and ensure the safety and effectiveness of this drug.

#### 4.4.3 Bumetanide

Bumetanide is a highly effective loop diuretic that acts by inhibiting NKCC1 and NKCC2, thereby reducing intracellular Cl^-^ concentrations and shifting the action of GABA from excitation to inhibition. Clinical trials involving children with ASD have shown that bumetanide not only alleviates the severity of ASD symptoms but also reduces GABA concentrations in the insular cortex. This reduction in GABA concentration is positively correlated with decreased scores on the Childhood Autism Rating Scale (CARS), confirming the clinical efficacy of bumetanide in mitigating core symptoms of ASD in young children. This improvement is also associated with a decrease in the GABA/glutamate ratio (159, 160). Additionally, it has a significant positive impact on the Social Responsiveness Scale (SRS) and Clinical Global Impressions - Efficacy (CGI-E) scores, but has no significant effect on sensory symptoms (161). However, the long-term therapeutic effects of bumetanide in children with ASD cannot be assessed based on current studies. Additionally, the side effects of bumetanide, such as hypokalemia and increased urination, cannot be overlooked. Furthermore, these trials have limitations, including small sample sizes, wide age ranges of participants, short treatment durations, and heterogeneity in patient treatment (162).

#### 4.4.4 Cannabidiol

Cannabidiol (CBD) is a non-psychotropic compound extracted from the cannabis plant and has been identified as a positive allosteric modulator of the GABAA receptor. Research emphasizes the importance of the endocannabinoid system (ECS) in neurotrophic factor signaling during fetal CNS development, regulating synaptogenesis, axon growth, neuronal cell fate, migration, and proliferation, as well as neuroprotection mediated by CB1R and BDNF, preventing neuronal death, and the ECS’s influence on dopaminergic systems (primarily through D2 receptors), GABAergic, and glutamatergic systems (163). It enhances endogenous CBD levels and GABAergic transmission, thereby improving the balance between inhibitory and excitatory neurotransmission (164). Studies by Pretzsch et al. have shown that in healthy adult males, CBD can increase GABA levels in the basal ganglia and dorsomedial prefrontal cortex, whereas these levels are often reduced in individuals with ASD. Additionally, cannabidivarin (CBDV) has been found to modulate the glutamate-GABA system in the basal ganglia without affecting the corresponding system in the frontal regions (165–167). An open-label study reported improvements in social communication in ASD youth treated with CBD-rich cannabis, with greater gains in those with more severe baseline symptoms. However, due to the lack of a placebo control and inherent biases of unblinded designs, these findings remain preliminary and insufficient to support clinical use (168). Despite showing promise in the treatment of ASD, CBD faces several challenges, including adverse effects, potential drug interactions, and regulatory issues related to the retail market for cannabis-derived products. To validate the efficacy and safety of CBD in ASD treatment, further clinical trials are needed, covering various doses and neurodevelopmental stages to understand its therapeutic potential (169).

#### 4.4.5 Mesenchymal stem cells

Mesenchymal stem cells (MSCs) are a type of multipotent stem cells with self-renewal capabilities, found in neonatal umbilical cord tissue. MSCs intervene in ASD through two mechanisms: First, they exert tissue repair functions by secreting cytokines, chemokines, and growth factors via paracrine effects. Second, they modulate immune responses. Studies have shown that certain types of nerve cellsin the brains of individuals with ASD, such as astrocytes, microglia, and Purkinje cells, exhibit abnormalities in morphology and number. MSCs can leverage their robust repair capabilities to restore damaged neurons and enhance synaptic function (170). Several clinical trials have demonstrated the potential of MSCs in treating ASD. A phase I open-label trial demonstrated that the infusion of umbilical cord tissue-derived mesenchymal stem cells (UC-MSCs) is safe and well-tolerated in children with ASD, with some patients showing improvement in ASD symptoms. Another open-label phase I/II clinical trial showed that the combined transplantation of cord blood mononuclear cells (CBMNCs) and UC-MSCs is more effective in improving ASD symptoms compared to the use of CBMNCs alone (171). Additionally, exosomes from MSCs (MSC-Exos) can suppress inflammation, restore key cellular functions,and initiate tissue repair and regeneration.An animal experiment reported that after delivering exosomes into the brain of VPA-induced autistic mice via intranasal administration, the mice exhibited improved self-grooming and reduced repetitive standing behaviors, increased time spent exploring unfamiliar mice, decreased levels of pro-inflammatory cytokines IL-1β, TNF-α, and IL-6, and increased levels of the anti-inflammatory cytokine IL-10 (172). However, the relationship between MSCs and the GABA signaling pathway has not yet been established. The existing clinical trials involving MSCs for ASD treatment are limited in number, and further experiments are needed to elucidate the mechanisms by which MSCs repair damaged GABA neurons, synaptic functions, and the GABA signaling pathway.

#### 4.4.6 Probiotic and fecal microbiota transplantation

**Figure 2:**
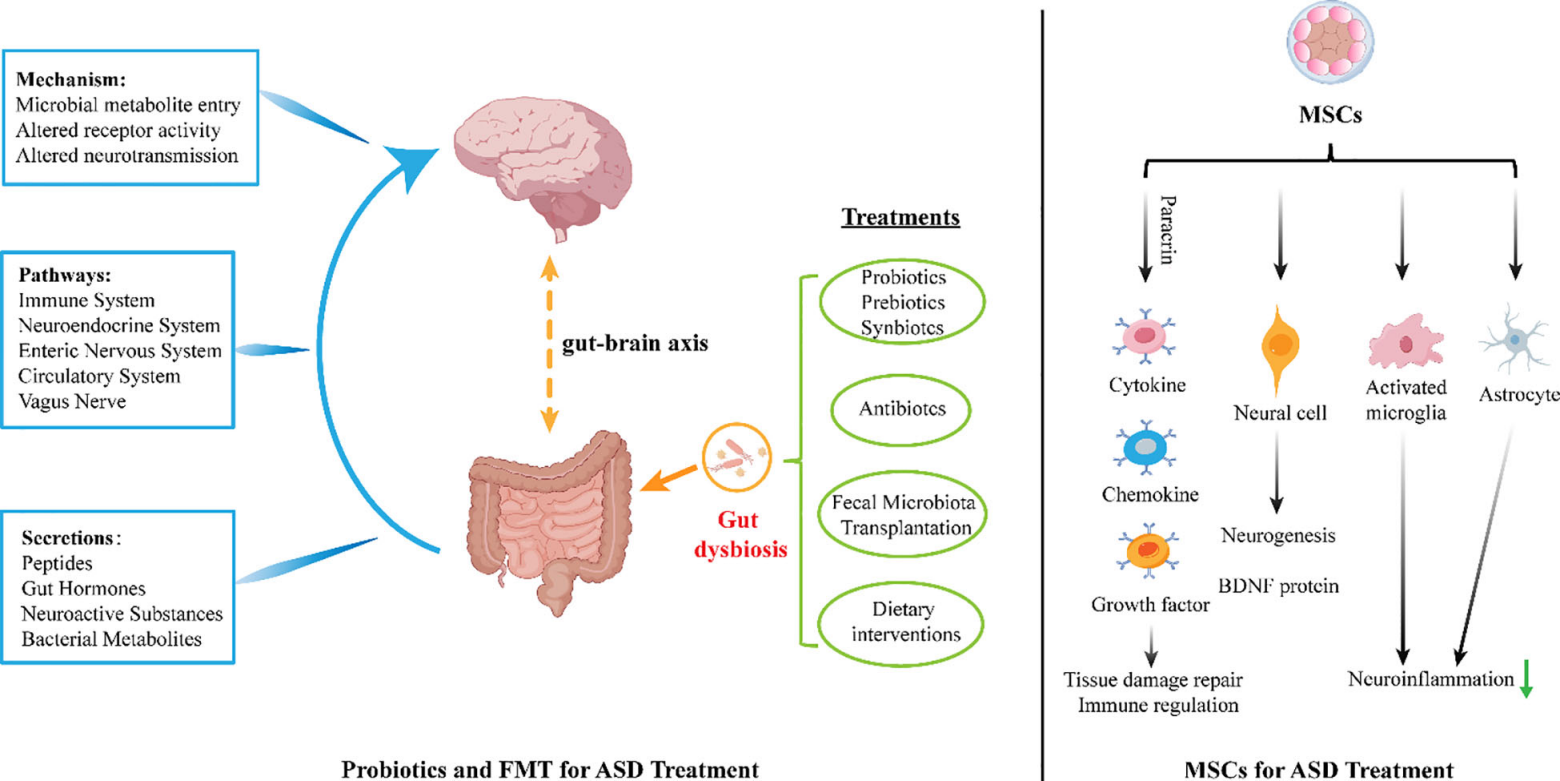
Gut dysbiosis can produce certain peptides, gut hormones, neuroactive substances, and bacterial metabolites through immune system, neuroendocrine system, enteric nervous system, circulation system and vagus nerve act on the brain, and treatment with probiotics and fecal microbiota transplantation can improve gut microbiota imbalance and alleviate symptoms in people with ASD. MSCs can exert tissue repair effects through paracrine effects by secreting cytokines, chemokines, and growth factors. Additionally, they can reduce neuronal apoptosis and produce neuroprotective effects by increasing the expression of brain-derived neurotrophic factor (BDNF) and reducing the number of hypertrophic microglia/macrophages. Furthermore, by reducing the activation of microglia and astrocytes and increasing the anti-inflammatory cytokine IL-10, MSCs can alleviate inflammatory responses.

Probiotic therapy and fecal microbiota transplantation (FMT) are interventions that modulate the microbiota-gut-brain axis. Individuals with ASD often exhibit significant gut microbiota dysbiosis, and the signaling molecules produced by these gut microbes can promote the synthesis of neurotransmitters (173, 174). For example, certain gut bacteria, such as *Bifidobacterium breve* and *Lactobacillus plantarum*, can participate in the synthesis and regulation of neurotransmitters like acetylcholine, dopamine, serotonin, and GABA, as well as their precursors, in specific gut cells such as enterochromaffin cells (175). Studies have shown that treatment with *Lactobacillus reuteri* or fecal microbiota transplantation can improve abnormal conditioned fear reflexes and GABAergic transmission in the amygdala of male *Cntnap4* knockout mice. Additionally, transplanting fecal microbiota from *Cntnap4* mice can restore conditioned fear reflexes and GABAergic transmission in these animals (176). Research by Bin-Khattaf et al. demonstrated that pure *Bifidobacterium* strains can improve core symptoms of propionic acid-induced autism in mice by restoring depleted GABA levels and upregulating GABA receptor gene expression (177). However, a recent randomized controlled trial by Meritxell showed that although a probiotic mixture of *Lactobacillus plantarum* and *Bifidobacterium breve* exhibited the ability to produce GABA, it did not significantly impact core ASD symptoms (178). In an experiment investigating fecal samples from children with mild ASD using multi-omics approaches, an imbalance in GABA metabolism was observed in the gut microbiota of children with mild ASD, characterized by an elevated GABA/Glu ratio, which could serve as a potential diagnostic marker for ASD. Additionally, the overabundance of Escherichia genus bacteria in the guts of ASD children was closely correlated with GABA metabolic disorders. Animal experiments further confirmed that overgrowth of E.coli may lead to social behavioral impairments (179). Currently, the mechanisms by which FMT and probiotics act on the GABA signaling pathway are not fully understood. Further large-scale, multicenter clinical trials are needed to elucidate their efficacy and safety. Moreover, the selection of microbial strains should be guided by integrative data from gut metagenomics, metabolomics, and neuroimaging techniques to optimize therapeutic outcomes (**Figure 2**).

#### 4.4.7 Other medications related to GABA

Acamprosate is a GABA analog. Early studies speculated that acamprosate might function by directly acting on GABAA receptors. However, subsequent *in vitro* experiments failed to confirm this hypothesis. For instance, in rat nucleus accumbens and cortical neurons, acamprosate did not significantly affect GABAA-mediated IPSCs. Additionally, acamprosate was able to partially reverse the increase in neuronal firing frequency and amplitude induced by the GABAA receptor antagonist bicuculline. Furthermore, administration of acamprosate led to increased GABA binding in the hippocampus and thalamus, suggesting that acamprosate might influence GABAA transmission by presynaptic inhibition of GABAB receptors (180). In a 10-week open-label trial of acamprosate in youth with FXS, treatment was associated with improvements in hyperactivity and social-communication measures, and no severe side effects were reported. However, the study was limited by its small sample size, open-label design, and inclusion of concomitant psychotropic medications, which makes it difficult to draw firm conclusions about efficacy. While these preliminary findings suggest potential benefit, larger randomized, placebo-controlled trials are necessary, and at present there is insufficient evidence to make strong recommendations regarding the clinical use of acamprosate or other GABAergic agents in ASD (181). Carnosine exerts its effects by reducing zinc and copper influx near GABA receptors, thereby indirectly enhancing GABA function, particularly in the frontal cortex (182). A double-blind, placebo-controlled trial by Chez et al. reported that L-carnosine supplementation (800 mg/day for 8 weeks) led to statistically significant improvements in receptive language, socialization, and behavior in children with ASD, as measured by the Receptive One-Word Picture Vocabulary Test and the Gilliam Autism Rating Scale (GARS), compared to placebo. Although these findings suggest potential benefits of GABA-related interventions—given carnosine’s proposed modulation of GABAergic activity and zinc/copper homeostasis—the mechanism remains unclear, and replication in larger, independent samples is needed (183).

Sodium valproate is used as an antiepileptic and mood stabilizer. Its role in regulating GABAergic transmission and its mechanism of action remain controversial. Sodium valproate does not directly bind to GABA receptors but regulates GABA concentration by increasing its synthesis and reducing its metabolism (184). Two small randomized, double-blind, placebo-controlled trials of divalproex sodium—a valproate compound—in individuals with ASD reported statistically significant improvements in core behavioral symptoms. One 8-week study (n = 13) found a large effect size (d = 1.616) in reducing repetitive behaviors, as measured by the C-YBOCS (185), while a 12-week trial (n = 27) showed significant improvement in irritability on the Aberrant Behavior Checklist-Irritability subscale and Clinical Global Impression-Improvement scale (186). Despite these promising signals, both studies were limited by small sample sizes and short durations, providing only preliminary evidence. Therefore, the efficacy of divalproex sodium in ASD remains uncertain and requires validation in larger, well-powered trials. In addition, exposure to valproic acid during embryonic development can interfere with the normal development of the nervous system. For example, administering valproic acid to pregnant mice during the embryonic stage can affect neurogenesis, migration, and differentiation in the embryonic brain, leading to abnormalities in neuronal structure and function, and subsequently resulting in ASD-like behaviors (187).

Riluzole, a recently developed drug initially used for amyotrophic lateral sclerosis, has been shown to directly affect postsynaptic GABAA receptors (188). In a 10-week, double-blind, placebo-controlled trial, adjunctive riluzole (vs. placebo) added to risperidone was associated with greater improvement in irritability, lethargy/social withdrawal, stereotypic behavior, and hyperactivity in children with ASD. However, this finding comes from a small study (n=40), and the effect of riluzole as monotherapy remains unknown due to the add-on design (189).

Flumazenil, a benzodiazepine antagonist, enhances GABA-induced GABAA-mediated currents while antagonizing the effects of benzodiazepine agonists on GABAA receptor responses. An experiment demonstrated that flumazenil has no beneficial effect on improving symptoms in ASD patients (190).

**Figure 3:**
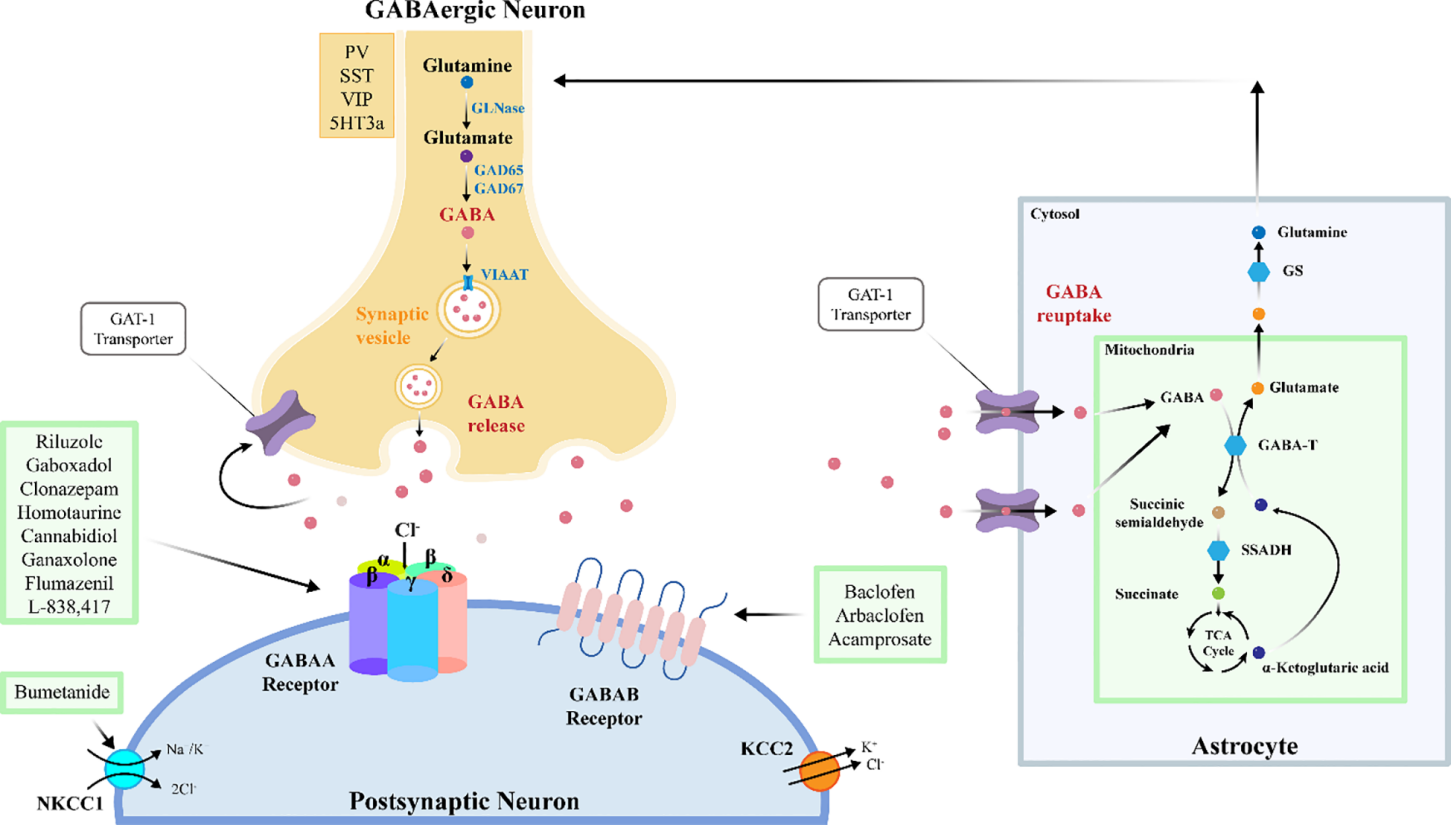
GABA synthesis, transmission, and metabolism. GABAergic neurons produce GABA via GAD56 and GAD57 (glutamic acid decarboxylase 56 and 57). GABA is transported into synaptic vesicles by VIAAT (Vesicular inhibitory amino acid transporter). Postsynaptic neurons can express GABA-A and GABA-B receptors, which mediate short-term and long-term effects of GABA on post-synaptic neuron activity. From the synaptic cleft, GABA is taken up by astrocytes via GAT (GABA transporter) to terminate signaling, and recycled through the GABA shunt and the TCA (Tricarboxylic acid) cycle. The glutamine thus produced is returned to GABAergic neurons. GS, Glutamine synthetase; GABA-T, GABA transaminase.

Despite promising preclinical data, clinical evidence for GABAergic drugs in autism spectrum disorder (ASD) remains inconsistent and largely inconclusive. While agents such as baclofen, arbaclofen, bumetanide, and cannabidiol have shown modest benefits in some trials, pivotal studies—particularly large placebo-controlled trials—often fail to demonstrate significant efficacy on primary outcomes. Findings are further limited by small sample sizes, short durations, add-on designs, and heterogeneous populations. Safety concerns, including hypokalemia, sedation, and teratogenicity, also restrict use. No GABA-targeting agent has regulatory approval for core ASD symptoms, and there are currently no strong clinical recommendations for their routine use. Future research should focus on biomarker-guided, stratified trials to identify responsive subgroups (**Figure 3**).

## 5 Conclusion

This study systematically reviews the role of GABA in the pathogenesis of ASD, highlighting the critical involvement of GABAergic systems in neurodevelopmental mechanisms and therapeutic strategies. By integrating findings from animal models, clinical imaging, and molecular biology, this work elucidates how dysfunctions in GABAergic interneurons (e.g., PV+, SST+, VIP+), GABA signaling pathways, and associated genes collectively disrupt the E/I balance in the brain, leading to core ASD symptoms such as social communication deficits, repetitive behaviors, and sensory abnormalities. Key discoveries include the impaired development and function of GABAergic interneurons, the complexity of GABA signaling disruptions (e.g., altered synthesis, transport, and receptor function), and the genetic-environmental interactions that amplify neurodevelopmental vulnerabilities. While GABAergic drugs (e.g., baclofen, bumetanide, homotaurine) show promise in preclinical models for ameliorating ASD-like behaviors, clinical translation remains limited by inconsistent efficacy and safety profiles. Current limitations include the heterogeneity of ASD, the challenges in detecting GABAergic abnormalities (e.g., reliance on invasive or low-resolution methods like MRI spectroscopy), and the need for more targeted drug development to minimize off-target effects. Future research should prioritize multi-omics approaches to dissect subtype-specific molecular mechanisms of GABAergic interneurons and their interactions with other neurotransmitter systems (e.g., glutamatergic, serotonergic pathways). The development of individualized biomarkers (e.g., GABA/Glu ratios via MRI) and precision therapies (e.g., gene editing, stem cell-based interventions) could refine diagnostic and treatment strategies. Cross-disciplinary collaborations and large-scale clinical trials are essential to validate long-term safety and efficacy, particularly in diverse ASD populations. In summary, GABAergic system dysregulation represents a central mechanism in ASD pathophysiology, offering both theoretical insights and therapeutic opportunities. Addressing current gaps through integrative, personalized approaches will be critical to advancing early diagnosis, mechanistic understanding, and targeted interventions. This work underscores the potential of GABAergic targets to transform ASD management while emphasizing the need for rigorous translational research to bridge bench-to-bedside gaps.
